# ALL IN HOW YOU LOOK AT IT: ATTITUDES, AFFECT, AND ABILITY IN THE ASSESSMENT OF SUBJECTIVE COGNITION

**DOI:** 10.1093/geroni/igae098.0023

**Published:** 2024-12-31

**Authors:** Emily Bower, Melissa Zammitti, Claudia Jacova Chenoweth

**Affiliations:** Chair; Co-Chair; Discussant

## Abstract

Subjective cognitive decline (SCD)—self-perceived persistent cognitive decline in the absence of objective cognitive impairment—is a risk factor for dementia. Despite the potential for SCD to aid in the detection of future dementia, differentiating trajectories of normative from non-normative decline among people with SCD is complicated for several reasons, including that self-perceived cognition is associated with socioemotional and general health factors in addition to objective abilities. Moreover, operational definitions of self-perceived cognition vary across the research literature and overlap with subjective aging. A robust understanding of the relationships among self-perceived cognition and aging, objective cognition, mood, attitudes, and health will strengthen the characterization and differentiation of SCD. This symposium will explore measures of perceived cognition and describe their relationships with mood, health, and aging attitudes among healthy older adults. The first presenter will describe the differential impacts of negative and positive affect on perceived memory difficulties, highlighting the complex relationships among depression, affective states, and subjective memory decline. The second presenter will expand on these findings by contrasting a measure of subjective memory decline with related measures of subjective memory and subjective age, and explore differential impacts of perceived health and objective memory. The third presenter will focus on perceived cognitive ability more broadly to describe its relationship with attitudes toward aging, depression, and objective cognition. The Discussant will compare and contrast findings with regard to operational definitions of self-perceived cognition and consider implications of the findings as they relate to emerging issues of clinical classification and management of SCD.

